# Codeveloping a Novel Intervention for People With Post‐COVID Condition: The Balance‐ACT Study

**DOI:** 10.1111/hex.70320

**Published:** 2025-06-08

**Authors:** Lily Felton, Michail Kalfas, Debbie Brewin, Carole Beckwith, Tasbiha Khan, Caroline Jolley, Nicholas Hart, Emma L. Duncan, Timothy Nicholson, Oliver Witard, Julie Moore, Alan Metcalfe, Gerrard F Rafferty, Trudie Chalder

**Affiliations:** ^1^ Department of Psychological Medicine, Institute of Psychiatry, Psychology & Neuroscience King's College London London UK; ^2^ Centre for Human and Applied Physiological Sciences, King's College London Faculty of Life Sciences & Medicine London UK; ^3^ Department of Respiratory Medicine King's College Hospital NHS Foundation Trust London UK; ^4^ Lane Fox Clinical Respiratory Physiology Research Centre London UK; ^5^ Lane Fox Respiratory Service London UK; ^6^ Department of Twin Research and Genetic Epidemiology King's College London London UK; ^7^ Guy's & St Thomas's NHS Foundation Trust London UK; ^8^ School of Life Course & Population Sciences, King's College London, Guy's Campus London UK

**Keywords:** acceptability, Long COVID, multidisciplinary care, patient outcomes, post‐COVID condition, psychological intervention

## Abstract

**Introduction:**

Some people experience persistent symptoms, such as fatigue, brain fog and breathlessness, long after the onset of COVID‐19 infection. This is known as the post‐COVID syndrome (PCS). We developed a unique and holistic psycho‐physiological intervention that integrates Acceptance and Commitment Therapy (ACT), a structured talking therapy, with principles of homeostasis. This aims to provide targeted support and treatment strategies for effectively managing the long‐term repercussions of the condition and improve patient outcomes.

**Methods:**

This empirical study was informed by theory and other research strands. These strands included a qualitative study of people with lived experience, a scoping review of interventions for fatigue (including rehabilitation) and insights from a patient and public involvement (PPI) group. The PPI group were actively involved in the development of the intervention, manuals and overall study management, ensuring it was relevant, ethical and aligned with patient preferences and needs.

**Results:**

The qualitative study uncovered the tangible contexts in which the intervention would be implemented, and the attraction of Balance‐ACT for those living with PCS. People living with PCS (*n* = 19) and health care professionals (HCPs; *n* = 12) provided overall endorsement for the intervention. Through an iterative process, their feedback, alongside input from the PPI group, informed the development of key materials, including a therapist manual, participant handbook, mindfulness recordings and an animation. Therapists were trained, and a novel fidelity measure was developed to ensure adherence to Balance‐ACT principles.

**Conclusion:**

We used an iterative approach to develop the Balance‐ACT intervention, which was acceptable to patients and HCPs. Subsequent research will examine the efficacy of Balance‐ACT.

**Patient and Public Contribution:**

This article represents work conducted as part of the Balance‐ACT project. People with the post‐COVID condition (PCC) were involved throughout all aspects of this study, in line with the National Institute for Health and Care Research framework, and contributed in several key ways. It ensured the research captured a diverse range of illness experiences. The study design was refined and addressed potential barriers to engagement. Patient‐friendly language was used to improve accessibility, making the study more inclusive. Additionally, the outcome measures were informed by patient input to enhance their relevance. Finally, dissemination was guided to ensure that the findings were both useful and accessible, using clear language in reporting and incorporating feedback from patient representatives on drafts to ensure clarity.

C.B. recorded the mindfulness exercises and was a core part of the management team throughout.

## 1 Introduction

Post‐COVID condition (PCC), also known as ‘Long COVID’, is characterised by persistence of symptoms at least 2 months after a SARS‐CoV‐2 infection, typically emerging within 3 months of initial infection [1]. Several terms have been used to describe this cluster of symptoms. The term ‘post‐COVID syndrome’ (PCS) is used by NICE in their guidelines [2]. However, we have chosen to use the term ‘PCC’ as it is more widely preferred internationally. Individuals with PCC may experience many symptoms, including but not limited to fatigue, brain fog and breathlessness. Prevalence of PCC varies widely internationally, with some estimates indicating around 5% of people who have had COVID‐19 continue to experience symptoms months after their initial infection [3]. In the United Kingdom alone, approximately 2 million people report suffering from post‐COVID symptoms, leading to reduced economic activity and chronic health conditions [4].

The putative pathophysiology of PCC is currently unclear, with much of the existing research derived from studies on posthospitalised patients. Ongoing research strives to elucidate various—and potentially interacting—mechanisms that contribute to the diverse symptoms experienced by individuals with PCC. Proposed mechanisms include immune system dysfunction and homeostatic imbalance. Evans et al. [5] found elevated pro‐inflammatory cytokines^1^ in a group of posthospitalised participants with severe PCC, which was linked to fatigue, brain fog and muscle weakness. This is consistent with research on obesity, in which there is chronic low‐grade inflammation and increased risk of severe COVID‐19 outcomes. Staatz [7] demonstrated that higher body mass index across the lifespan increases vulnerability to acute illnesses.

In response to the virus, the body's threat responses trigger symptoms such as fatigue, brain fog and distress, reflecting the interconnected nature of disturbances that emerge as part of the body's ongoing response [8]. Whilst there is uncertainty around the exact causes and mechanisms underpinning the diverse range of symptoms, there is a pressing unmet need for holistic interventions that target the varied symptoms and promote quality of life (QoL) for people living with PCC. Our intervention was developed for individuals in outpatient or community settings within the National Health Service (NHS), addressing their unique needs and experiences during and after acute infection.

In common with many chronic illnesses, support for individuals with PCC is often insufficient and/or inaccessible. Several studies report widespread frustration with available health care support and unclear guidance among people with PCC, leading many to seek support elsewhere [9, 10, 11, 12]. Qualitative studies have highlighted the nuanced needs of people living with PCC. Researchers consistently highlight that individuals with PCC need a multifaceted approach to address the range of needs and challenges they face, emphasising the importance of personalised care that considers individuals' unique experiences and includes educational resources and practical tools to aid the daily management of PCC [13, 14]. Structured and semi‐structured interviews have revealed a strong preference for multidisciplinary treatment addressing both physical and psychological symptoms [15, 16].

As with many long‐term conditions, treating underlying pathology directly can be challenging, and, given the unknown mechanism(s) for PCC, indeed not possible. Irrespective of the ultimate cause, it is crucial, therefore, that additional non‐pharmacological approaches to treat PCC are evaluated. Focusing on improving QoL despite ongoing symptoms may represent the most practical and effective strategy. Given the complex nature of PCC, psychological/behavioural interventions can address interacting psychological and physiological processes. For example, mindfulness may facilitate autonomic regulation, whereas regularising activity and sleep may promote homeostasis and help regulate the body clock.

Although there is growing recognition of the need for interventions to support individuals with PCC, few programmes have been developed specifically for this population. A recent systematic review [17] concluded that combined physical and mental health rehabilitation programmes may improve symptoms such as fatigue and QoL in individuals with PCC [18, 19]. Physical rehabilitation approaches [20, 21] and neurological retraining programmes [22] have shown promise in addressing fatigue and physical function. The REGAIN programme [19], which offered group‐based psychological support alongside physical rehabilitation, demonstrated significant and sustained improvements in health‐related QoL, highlighting the value of integrated, holistic care for PCC recovery. Similarly, Derksen et al. [23] combined mindfulness, pacing and education, which showed promise in supporting symptom management. Cognitive–behavioural therapy (CBT) has also shown effectiveness in symptom reduction, particularly severe fatigue [24], supporting the value of tailored, therapist‐supported interventions. Our approach uses a different theoretical model that integrates hypothesised physiological and psychological mechanisms of change in a complex intervention we call Balance‐ACT to target both symptoms and broader functional and emotional outcomes.

The development of Balance‐ACT was informed by Acceptance and Commitment Therapy (ACT), an empirically based talking therapy that facilitates behaviour change and aims to improve life experiences and overall well‐being by increasing psychological flexibility [25]. It is rooted in the principles of ACT theory, emphasising the ability to adapt to life's challenges and demands, shift perspective and balance competing desires and needs. Psychological flexibility has been found to mediate change in a range of outcomes across both physical and emotional health conditions [26]. ACT focuses on helping individuals accept what they cannot change directly (i.e., symptoms), encouraging meaningful actions consistent with personal values, whilst increasing flexibility of coping responses. It is person‐centred, compassionate and grounded in empathy, addressing the varying difficulties faced by patients, with the ability to work with clinical and socio‐cultural variability. ACT uses mindfulness to help individuals *notice* difficult thoughts, feelings and physical sensations without trying to change them. Some existing programmes include mindfulness and acceptance strategies, but the evidence is limited, and more research is needed to confirm their effectiveness for PCC. Although direct evidence for ACT in PCC is lacking, there is evidence of its effectiveness in chronic pain [27] and other long‐term conditions [26]. Indeed, we found a relatively small number of brief therapy sessions to be helpful for people with muscle diseases [26, 28, 29], suggesting that ACT may provide a beneficial framework for improving QoL for people with PCC.

Balance‐ACT is explicitly grounded in ACT principles to enhance flexibility and alignment with individual values. Additionally, it aims to restore homeostasis, which is often disrupted in COVID‐19 [30, 31]. The name ‘Balance’ has been deliberately chosen to reflect and play on multiple concepts, including the regulation of physiological functioning through strategies such as optimising sleep and activity levels, which aim to restore balance to the body and mind. We simplified the six core ACT processes [32] to address the difficulties of PCC patients. Participants were encouraged to ‘open up’ to all experiences, ‘be present’ in the moment and ‘do what matters’ by adjusting priorities based on their needs and recovery stage. We avoided traditional ACT terms like ‘cognitive fusion’ and used simpler phrases such as ‘being stuck’ or ‘caught up’ to prevent misconceptions that symptoms were ‘all in the mind’, which could cause distress and a feeling of being dismissed by health professionals.

### 1.1 Overarching Aim

This manuscript will describe the process by which the intervention was codeveloped and refined, with several components of research carried out alongside one another and integrated through an iterative approach. This work drew on ACT theory, knowledge of homeostasis and patient input to develop the manuals and associated resources in an accessible way. The incorporation of a patient and public involvement (PPI) group aligns the intervention with patient preferences and needs, engendering a more impactful and sustainable approach to care. The overarching aim was to develop an individually tailored intervention acceptable to individuals with PCC and suitable for people with a wide range of difficulties.

In the following sections, we first outline the methods used to design and deliver the Balance‐ACT intervention, with the methods organised according to the intervention's key strands. Results are subsequently presented in alignment with these strands, illustrating how the intervention has been refined in response to ongoing feedback and evaluation.

## 2 Methods

### 2.1 Intervention Development Components

**Figure 1:**
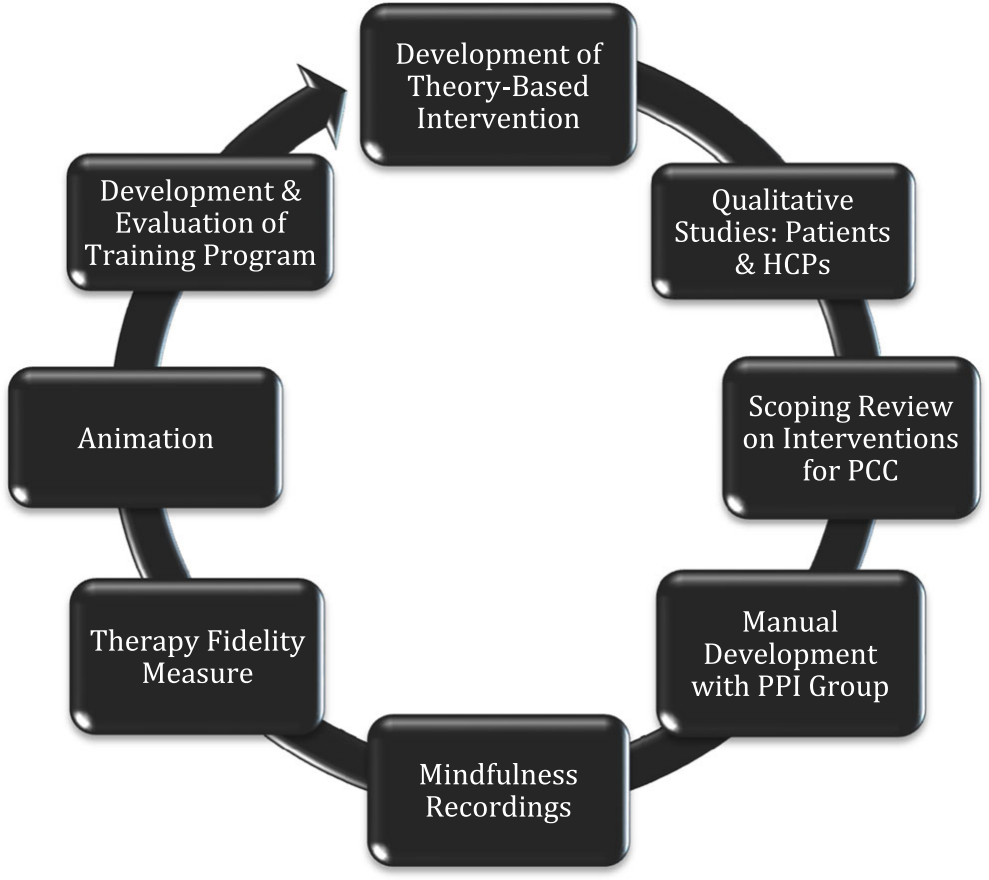
Intervention development components.

We present the development of ACT across multiple components of work, as illustrated in Figure 1.

#### 2.1.1 Component 1: Development of a Theory‐Based Intervention

The Balance‐ACT intervention for PCC is rooted in the principles of ACT [33] and adapted to help people manage both physical and mental symptoms while restoring homeostasis. ACT in this context promotes psychological flexibility and supports people to accept the challenges associated with unpredictable, disruptive symptoms, while engaging realistically in meaningful activities.

The following patient accounts illustrate this:

1. One patient described how being mindful in the shower allowed her to enjoy the warmth of the water, rather than focusing on the pain.
2. Another shared how being present without judgement helped prevent rumination on pre‐COVID abilities.

We incorporated the six key therapeutic processes within the ACT Hexaflex Model [33] and merged them with an adaptation of the three‐pillar model [32]:

1. *Open* (acceptance and defusion^2^). Opening up to, rather than controlling, private experiences; personal history; symptoms or attitudes/behaviour of others. This includes recognising and relating differently to thoughts, symptoms and feelings.
2. *Aware* (present focus, observing self). Shifting attention flexibly to the present moment. Involves awareness of both internal and external experiences, and the ability to step back and gain perspective.
3. *Active* (values, committed action). Identifying what matters personally and taking actions towards values‐based goals, even in the presence of unwanted symptoms, thoughts and feelings.

Balance‐ACT helps people with PCC embrace life to the full, despite discomfort, shifting focus from fixing difficulties to pursuing what matters—aligned with their values and aspirations—in a balanced way.

**Figure 2:**
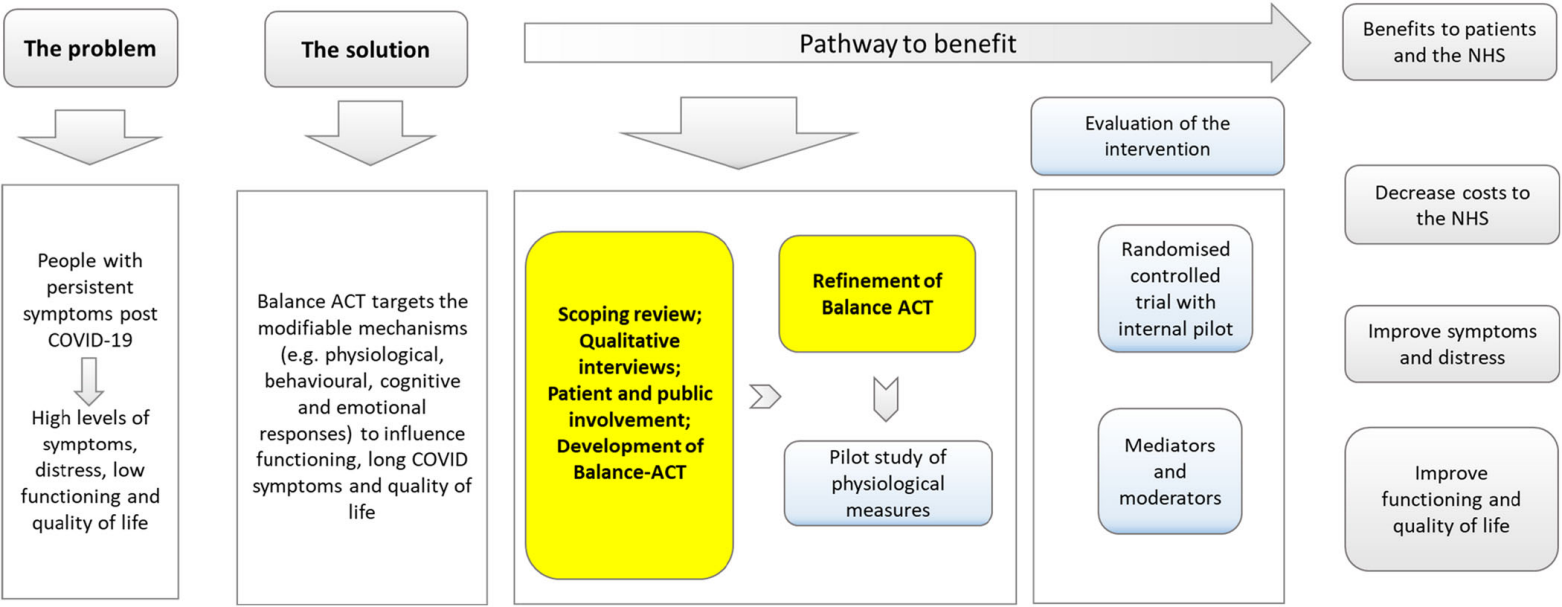
Development of the Balance‐ACT intervention and the potential benefits.

Figure 2 illustrates the development process of Balance‐ACT, showing the stages designed to optimise the approach.

**Figure 3:**
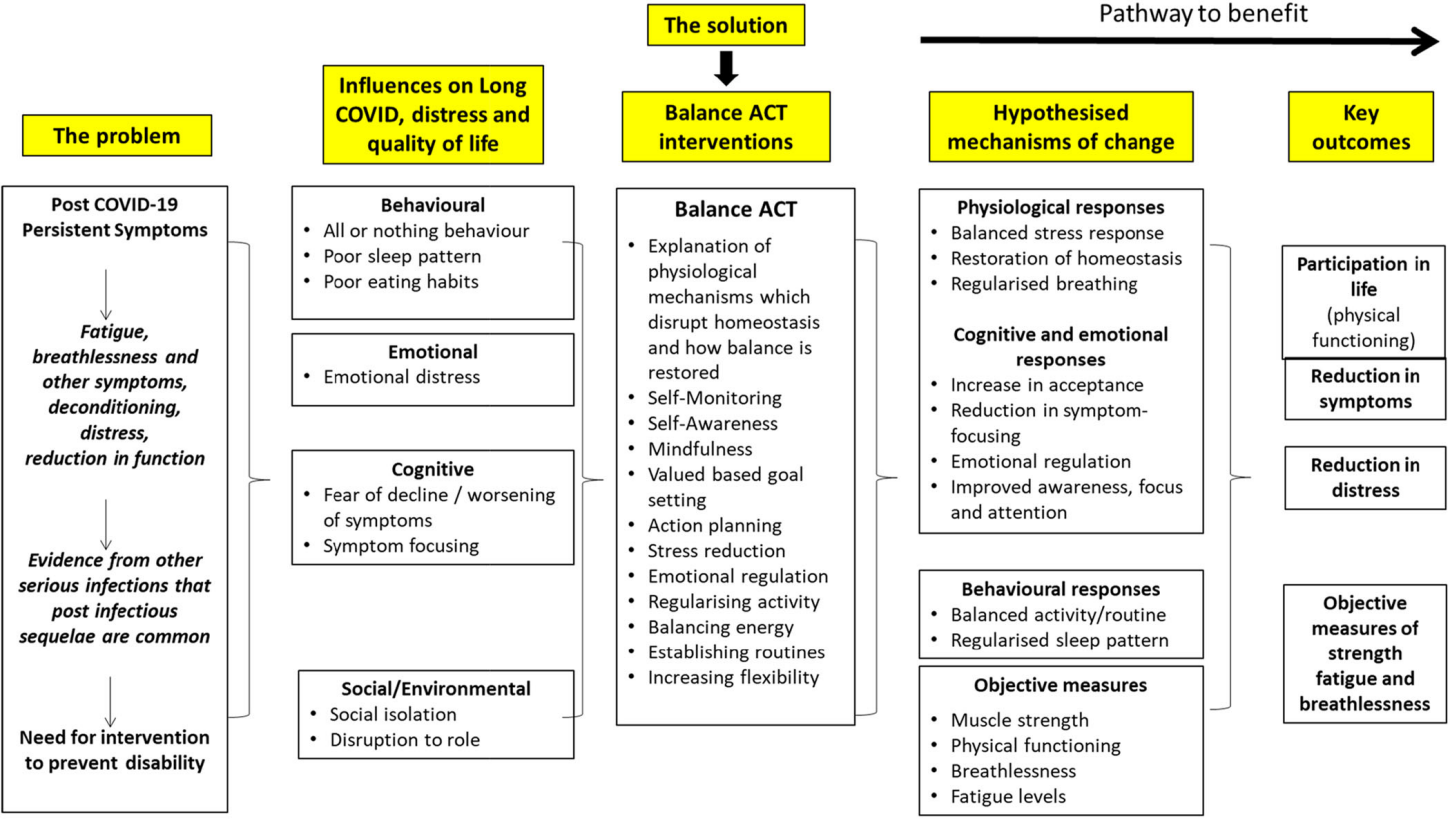
Logic model for Balance‐ACT. The model depicts hypothesised mechanisms of change which may be linked to improved treatment outcomes.

Theory‐based logic modelling was used to align existing evidence and core intervention components with proposed strategies for behaviour change and intervention outcomes, aiming to address homeostasis and help patients manage symptoms effectively. The main components of our proposed logic model for Balance‐ACT are shown in Figure 3. The development of the LOGIC model was based on the core principles of ACT theory, which emphasises acceptance, psychological flexibility and behaviour change. The model addresses the complex interplay of symptoms, influences, solutions and outcomes, underscoring the importance of a holistic understanding of patient difficulties and the necessity for integrated solutions to support long‐term health. Our hypothesised mechanisms of change were influenced by the theory of ACT, as well as behaviour change interventions focused on achieving homeostasis, for example, adopting a good sleep routine.

#### 2.1.2 Component 2: Qualitative Studies: Health Care Professional and Patients

After developing a preliminary outline of the intervention, we conducted qualitative interviews with individuals living with PCC and with health care professionals (HCPs), soliciting their perspectives on what we proposed. The first part of the qualitative interviews explored the experiences and needs of people with PCC and is published elsewhere [34]. The findings presented here represent the second aim of the qualitative interviews, which focused on the acceptability and feasibility of Balance‐ACT. This project received ethical approval by the King's College London Research Ethics Committee (KCL REC; MRA‐21/22‐33455).

Nineteen individuals with PCC, who had consented to be contacted for future research in PCS (13 females, 6 males), along with 12 staff working within the NHS (7 females, 5 males), were recruited. Participants provided written consent, which was countersigned by the study researcher before participation. Semi‐structured qualitative interviews were conducted remotely, via Zoom or Microsoft Teams, and lasted approximately 60 min. Interviews began with a short description of the context and rationale of the proposed intervention by a researcher. Subsequent questions focused on their understanding of the intervention, its perceived value, any potential challenges they foresaw arising, as well as ways to deliver it more effectively. The interviews comprised open‐ended questions following an interview guide (see Table A1 in the Appendix). The interviewer adapted subsequent questions based on participants' responses.

Once anonymised, transcripts were uploaded and coded on NVivo (version 12) software. A coding framework was developed through successive refinements. The interviews were transcribed verbatim and examined inductively using reflexive thematic analysis [35]. Preliminary and final themes were discussed with the team, including the PPI group, before being drafted and reviewed.

#### 2.1.3 Component 3: Scoping Review on Interventions for PCC

A rapid scoping review was conducted to summarise current evidence on non‐pharmacological interventions for PCC. A systematic search was conducted on multiple databases, including KCL Library, Google Scholar and EMBASE, using predetermined search terms, including ‘Long COVID’, ‘PCC’, ‘fatigue’ and ‘non‐pharmacological interventions’. Searches were conducted for articles published between January 2008 and December 2023. Given the limited rigorous evidence on PCC‐specific interventions, we also included studies on non‐pharmacological interventions for conditions with symptom overlap, such as chronic fatigue syndrome and chronic respiratory diseases. Studies were included if they reported on adult populations (over 18 years) experiencing either PCC or related symptoms and focused on non‐pharmacological interventions. This approach enabled a broader synthesis of transferable interventions, whilst acknowledging condition‐specific factors. Studies were excluded if they were protocols, commentaries or not written in English. Eligibility of full texts was assessed by one reviewer and verified through discussion with the corresponding author. Data were extracted on sample sizes, types of interventions and key outcomes.

#### 2.1.4 Component 4: Manual Development With PPI Group

Although ACT has shown efficacy for pain and other long‐term conditions, no trials have explored its effectiveness for PCC. Several studies have used rehabilitation and self‐management for PCC, informing our choice of ACT as an overarching approach. The intervention was collaboratively developed by the principal investigator (PI), research team and PPI members. It was further shaped by the qualitative study exploring the experiences, needs and perspectives of people with PCC [34], and by feedback on its acceptability and feasibility.

**Figure 4:**
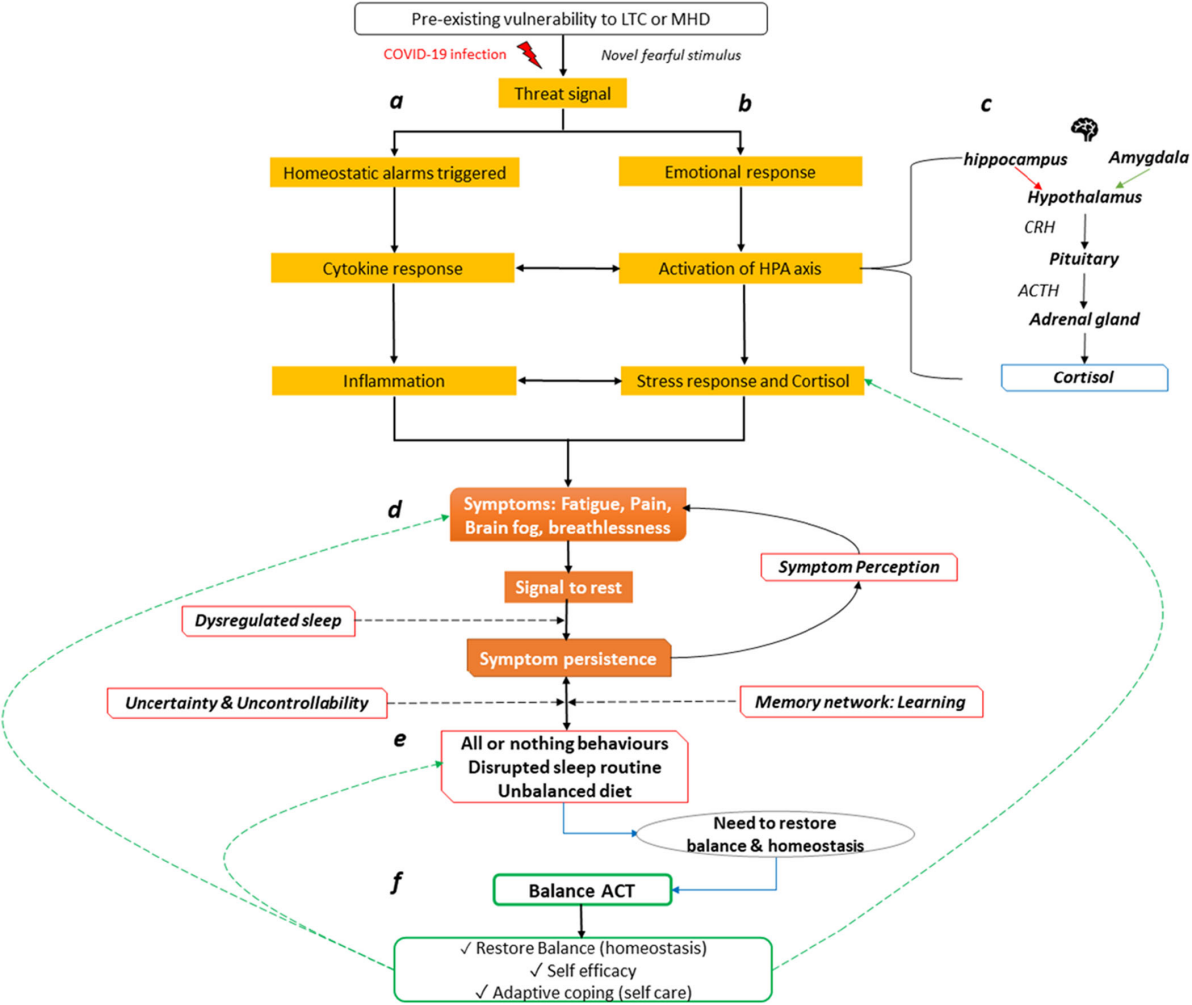
Balance‐ACT theoretical model. LTC, long‐term condition. *Note:* Different colours and letters have been used in the diagram to indicate the various processes included in the model (e.g., a, the physiological response; b, the emotional response; c, HPA axis; d, symptoms and initial responses; e, behavioural responses; f, the interventions which aim to restore balance).

The Balance‐ACT model, grounded in theory, expounds the longitudinal, interconnected nature of physiological, psychological and behavioural processes (Figure 4). COVID‐19 acts as a profound health threat, activating the body's alarm systems, triggering immune activation, inflammation and alterations in the hypothalamic–pituitary–adrenal (HPA) axis. These physiological imbalances, intended to protect the body, can become dysregulated perpetuating a cycle of physical and psychological symptoms. Such symptoms often lead to behavioural patterns, like all‐or‐nothing behaviours, which further reinforce the imbalance. The model provides a robust foundation for an intervention aiming to restore equilibrium with targeted, cross‐domain regulation.

Research on ACT and its efficacy in enhancing QoL for individuals with physical symptoms was reviewed before intervention development. ACT's focus on acceptance, psychological flexibility and values‐based living made it an appropriate approach for addressing PCC‐related challenges. This informed the preparation of a therapist manual and a patient handbook, created to increase flexible use and support improved QoL.

ACT treatment protocols for persistent physical symptoms and long‐term conditions informed some of the exercises in the participant handbook, which are referenced within. The handbook outlined the framework for the 10‐session, individually tailored intervention.

The PPI group comprised seven members, five on the advisory group, all recognised as experts by experience. They contributed to the qualitative research, provided input on the intervention development and provided written and verbal feedback on the manual. One member is part of the trial steering committee, and another (C.B.) sits on the Trial Management Group (TMG). The manual, participant handbook and training programme were iterative, involving continuous refinement and feedback. All documents were circulated in the TMG and shared with C.B., who provided key input and attended training sessions to advise on participant experience. Ethical approval was granted by KCL REC [23/LO/0941].

#### 2.1.5 Component 5: Mindfulness Recordings

Mindfulness skills are fundamental to ACT and, therefore, the Balance‐ACT intervention. Mindfulness improves homeostatic mechanisms, such as stress reduction and sleep [36], as well as attention and cognitive function [37]. Mindfulness promotes a focus on the present moment, acceptance, emotional regulation and psychological flexibility [38], with potential to address both psychological and physical aspects of PCC. One PPI member found it beneficial in their own PCC journey and supported its use in the intervention.

Mindfulness practices were specifically scripted and gradually refined to meet patient needs. A total of six mindfulness exercises were created by the therapist trainer (D.B.) and audio recorded by C.B., each ranging from 3 to 5 min. Exercises were simplified into three core skills, encapsulated by the mnemonic ‘NET’, which stands for Notice (be present and aware and describe or name experiences), Embrace (be open, compassionate, willing, curious and make room for all experiences) and Take a Helpful Action (widen the focus and perspective and take values‐guided action). The mnemonic maps onto the simplified ACT model (refer to Figure 4). See Table A4 in the Appendix for exercise overview.

#### 2.1.6 Component 6: Therapy Fidelity Measure

Treatment fidelity denotes the degree to which an intervention was implemented as intended [39]. A lack of treatment fidelity raises concern about the validity of a study and undermines any conclusions regarding a treatment effect. This poses a threat to the study's internal validity, potentially hindering future replication efforts aimed at establishing its external validity. There is no widely applicable fidelity measure for ACT, and the most commonly used measure has not been comprehensively corroborated [40]. Thus, we adapted a recently developed ACT fidelity measure (ACT‐FM) by O'Neill et al. [41], in conjunction with purposely developed items specific to Balance‐ACT.

#### 2.1.7 Component 7: Animation

To enhance delivery and engagement of the intervention, a 10‐min animation was developed. Simplifying complex neurophysiological processes and avoiding medical jargon was key, particularly for participants with PCC experiencing ‘brain fog’, fatigue and/or emotional distress [42, 43, 44]. The animation included key information about physiological and psychological functioning. The therapist trainer worked alongside the trial management team and an artist to script an animation explaining homeostasis, the autonomic nervous system and ideas to achieve mind–body balance within Balance‐ACT.

The animation was broken into sections covering physiological and psychological stress, the influence of thoughts and behaviours and the importance of balanced routines. A simple and engaging visual style was used, with subtitles for accessibility. Pacing was adjusted to support understanding, and it was formatted in a high‐resolution video file, shared via a private YouTube link. The development process was iterative, incorporating feedback from PPI, HCPs and the TMG. The animation was approved by REC.

#### 2.1.8 Component 8: Development of Training Programme

The training programme covered key topics for delivering Balance‐ACT, with an ACT‐informed, process‐based approach embedded throughout. It included content on physiological mechanisms, sleep, energy management and breathing techniques.

Training was delivered via interactive workshops over 5 days to three therapists (one occupational therapist and two physiotherapists), all experienced in PPS or PCC. Sessions included experiential exercises, presentations, video clips and a comprehensive therapist manual. After core training, therapists reviewed the manual and participant handbook before practising in live sessions. Initial sessions were closely supervised, with feedback provided using the fidelity measure. Thereafter, weekly group and ad hoc supervision was provided to ensure adherence. Appendix Table A2 outlines the training. The therapist manual also included session delivery tips and key objectives. A booster session was added 1‐month post‐training to reinforce learning.

Therapists' knowledge and confidence were assessed at registration, post‐core training, and post‐booster via a 28‐item self‐report questionnaire: 14 true/false items on PCC and ACT knowledge, and 14 Likert‐scale items on confidence (1 = *not confident*, 5 = *completely confident*). The aim was to evaluate training effectiveness and identify areas for improvement.

The following section presents the findings from each component of the intervention development process described above: key themes from interviews with patients and HCPs, insights from the scoping review, feedback informing the manual, mindfulness materials and animation, the fidelity measure and the therapist training programme.

## 3 Results

### 3.1 Components 1 and 2: Qualitative Studies: HCP and Patients

The interviews included 31 participants (19 females, 12 males), consisting of patients with PCC, HCPs and PPI members, aged 26–78 years. Most were White British (*n* = 25); full demographics are in Appendix Tables A3.1–A3.3.

This section summarises key themes from patients and HCPs regarding perceptions of the intervention and factors influencing its real‐world effectiveness: ‘Acceptability’, ‘Flexibility of Intervention Delivery’ and ‘Barriers and Suggestions for Implementing ACT Intervention’. Supporting quotes appear in Appendix Table A4.

#### 3.1.1 Theme 1: Acceptability

##### 3.1.1.1 Potential Benefits/Usefulness

Most participants responded positively to an ACT‐based intervention for PCC, acknowledging it as a strong fit for the diverse needs of this population. Patients and HCPs attested to the benefits of incorporating Balance‐ACT into the PCC care pathway, particularly given limited existing support. HCPs familiar with ACT praised its holistic approach in addressing patients' physical and psychological needs. The focus on exploring values was especially well‐received, with acceptance viewed as crucial when dealing with long‐term conditions.

Additionally, the patient population was noted to be particularly motivated, suggesting strong potential for engagement.

##### 3.1.1.2 Perceived Safety

Safety was a key concern. Patients consistently described the design as fostering a sense of psychological safety, which supported engagement and progress. HCPs considered the intervention low risk.

##### 3.1.1.3 Intervention Makes Sense

Participants felt the intervention aligned with their expectations, daily needs and values. One patient emphasised the content's relevance; however, others were not familiar with scientific terms like ‘homeostasis’, underscoring the importance of accessible language.

HCPs valued the inclusion of physiological and psychological explanations, noting this enhances patient awareness of their own needs, supporting engagement.

Tailoring the intervention to individual values and needs, and allowing flexible pacing, were seen as crucial to empowering participants and promoting autonomy.

#### 3.1.2 Theme 2: Flexibility of Intervention Delivery

##### 3.1.2.1 Accessibility

Views on the remote components of the intervention were mixed. Several HCPs appreciated its convenience and reach, whilst others felt that it might hinder engagement, particularly for older individuals who may find navigating online platforms challenging. Flexibility in delivery options was seen as key to engagement and retention.

#### 3.1.3 Theme 3: Barriers and Facilitators to Implementing ACT Intervention

##### 3.1.3.1 Heterogeneity/Catering to Participants With Different Needs

Symptom variability posed a concern for the effectiveness of a complex intervention like Balance‐ACT. The heterogeneity of symptoms experienced by patients presented significant challenges in delivering a one‐size‐fits‐all approach. Patients noted that no one experience of PCC is the same and developing an approach that caters for this will be difficult (‘I suppose the challenge is, but it's always the case, I guess with any health thing, is that our all of our experiences of Long COVID are very different.’). This view was echoed by several HCPs, who said that some patients struggle more with physical symptoms, whereas others are more affected by the psychological impact. This observation presents a challenge, as certain types of support may be relevant for some individuals but not for others. Hence, addressing symptom heterogeneity was regarded as key to meeting the needs of a diverse patient pool effectively.

##### 3.1.3.2 The Importance of Acknowledging the Reality of the Symptoms

Several participants worried that psychological approaches may not resonate with some individuals, particularly when used for physical symptoms like fatigue. HCPs agreed, suggesting some will embrace the idea of holistic treatment whilst others may be more medically focused.

Several HCPs noted that grounding the intervention in evidence could enhance understanding and engagement. HCPs emphasised the importance of giving patients space to express their thoughts and concerns, and allowing time for individuals to try different exercises independently, was vital for personalising the intervention, particularly given previous experiences of feeling dismissed by HCPs.

##### 3.1.3.3 Acceptance

A prominent challenge was the emotional difficulty in accepting symptoms. There was a prevailing sense of grief over the lost functioning, which exacerbated the struggle to come to terms with their current reality. Moreover, patients reported that judgement from others, within the medical system and society in general, compounded the difficulty of acceptance. HCPs emphasised that this not only impacted well‐being but also limited treatment engagement. Many felt that Balance‐ACT could help address this challenge by providing supportive tools for managing change.

##### 3.1.3.4 Creating Engaging Sessions

Patients wanted sessions to support both mental and physical well‐being, suggesting guidance on physical activity and energy management would be useful. Sleep and nutritional advice tailored to PCC were also seen as important for resilience, equipping individuals with a holistic set of tools to manage well‐being.

Patients recommended sessions be 45–60 min, allowing for meaningful engagement without fatigue. A 10‐session format was viewed as realistic and manageable, providing a clear, time‐limited framework, supporting learning and practice.

### 3.2 Component 3: Scoping Review on Interventions for PCC

The rapid scoping review examined a range of randomised controlled trials, meta‐analyses and systematic reviews, identifying a range of non‐pharmacological interventions for PCC and related symptoms. These included CBT [24], mindfulness‐based interventions [45], exercise therapy [46] and rehabilitation programmes [47]. Many studies reported positive outcomes, such as enhanced mental health indicators and reductions in fatigue, depending on the intervention. However, a significant number were excluded due to small sample sizes and short follow‐up periods. Additionally, there was a lack of research on ACT in the context of PCC, despite its proven effectiveness in other long‐term conditions [28]. The most relevant findings are summarised in the Appendix (Table A5) based on criteria such as intervention type, population and outcomes measured. The findings highlight the need for a holistic approach that addresses both the physical and psychological dimensions of PCC using larger sample sizes and long‐term follow‐up to determine the efficacy of non‐pharmacological approaches.

### 3.3 Component 4: Manual Development With PPI Group

Two distinct manuals were developed: a participant handbook and a therapist manual. The participant handbook contains multiple sections that guide participants through the intervention process, incorporating detailed information on ways of implementing key components and self‐reflective exercises to achieve the objectives of the intervention. Table A6 in the Appendix summarises the Balance‐ACT intervention patient‐facing content. The therapist manual describes the phases of the intervention with structured guidance for effective delivery. Table A7 provides a summary of the contents of the final version of the therapist manual.

The PPI group were given time to read, reflect, discuss and provide feedback on the content and style of writing. Table A8 charts the changes made to the participant handbook, therapist manual and training following feedback from the PPI group, TMG and research ethics committee, and how this translated into changes in the materials as part of the iterative development process. A key challenge was to reduce the size of the documents and organise them into manageable sections which were appealing to read, contained all the necessary information for this complex condition and did not appear too overwhelming for participants.

### 3.4 Component 5: Mindfulness Recordings

Overall feedback indicated that audio clips would be a useful addition to the mindfulness exercises in the participant handbook and could be used for home practice. Therefore, the therapist trainer developed eight transcripts of mindfulness practices, which were audio recorded by C.B., who paced the delivery of these practices with insight into how they would be most effectively received by the target audience.

### 3.5 Component 6: Therapy Fidelity Measure

We developed a novel fidelity measure, created through a multiphase process. Following a comprehensive review of existing fidelity measures and input from various field experts, a final instrument consisted of three subscales (see Table A9). A continuous rating scale was used rather than dichotomous scoring, in keeping with the system used in the Revised Cognitive Therapy Scale [48]. The rating scale emphasised items consistent with ACT principles rather than ACT‐inconsistent items. The measure effectively captured key aspects such as therapist stance, open response style, aware response style and engaged response style, suggesting its potential utility for evaluating and improving therapist adherence to Balance‐ACT principles in clinical practice. A total score was calculated by adding the subscale scores together. This newly developed scale will be utilised to assess the trial therapists' competency and fidelity to the intervention within the study. Throughout the delivery of Balance‐ACT sessions, we intend to adhere to the Perepletchikova et al. [39] checklist on the appropriate procedures required in the assessment of treatment integrity. Utilising a manualised therapy approach with all therapist–participant sessions audio recorded will also help assess fidelity.

### 3.6 Component 7: Animation

The development of the animation involved a comprehensive process comprising multiple phases: initial research, scripting, filming and editing. Feedback from internal stakeholders was continuously integrated, with adjustments and iterative refinement throughout, to improve clarity and engagement. PPI members underscored the difficulties of relying exclusively on written materials, highlighting the diversity in learning styles across different populations. It was therefore considered important that video clips included illustrations, audio descriptions and words, which would increase options and be an important addition to the materials. Final adjustments were made to address this feedback, ensuring the animation catered to all learning styles.

### 3.7 Component 8: Development of Training Programme

Feedback from the PPI group, TMG and REC was collected as part of an iterative process, as shown in Table A5. The pre‐ and post‐training analysis showed an increase in knowledge scores for all three therapists from registration to the end of training. Mean knowledge scores were 11.00 (SD = 1.00) at registration, and 12.33 (SD = 1.53) at the end of training and remained high at the booster session.

Mean confidence scores were 41 (SD = 6.08) at registration, 52.33 (SD = 4.04) at the end of training and 52.33 (SD = 5.03) at booster training.

These findings indicate an increase in confidence following training; however, the small sample size (*n* = 3) precluded formal statistical analysis. The small sample size was intentional, as the programme aimed to train therapists who would deliver the therapy in the trial. Efficacy will be assessed in a future RCT.

## 4 Discussion

Using a research‐focused approach, alongside people with lived experience of PCC, we codesigned an intervention for PCC called Balance‐ACT. Once we had a blueprint for what the intervention might look like, we conducted qualitative interviews with patients and HCPs and explored their perspectives on the acceptability and feasibility of the approach. We were interested in their feedback on the theory and content of the intervention, and the extent to which it made sense. We recognised the diverse specialties in which patients were being assessed and deemed it important to explore whether HCPs' perspectives were consistent with those of patients.

Three key themes emerged: ‘Acceptability’, ‘Flexibility of Intervention Delivery’ and ‘Barriers and Suggestions for Implementing ACT Intervention’. Acceptability was not described as the passive process of giving up, but rather as an active process of embracing thoughts, feelings and physical sensations/symptoms without trying to avoid, ‘fix’ or control them. This may be counter‐intuitive; however, ACT offers individuals a variety of ways to manage difficult situations whilst taking action towards what matters most to them. Participants viewed this definition of acceptance positively and acknowledged its importance in their patient journey [34]. Similarly, research in other long‐term conditions reports an association between acceptance and better QoL [49, 50]. Participants in our study highlighted the importance of the flexibility of the intervention, especially as PCC is highly heterogeneous. This observation is consistent with the ACT framework, which highlights the importance of flexibility in responding to experiences based on context and individual needs, ultimately fostering flexible behavioural responses—prioritising actions that align with personal values and supports well‐being.

The development of the Balance‐ACT approach was informed by findings from the qualitative interviews. We ensured the intervention was grounded in the needs and expectations of people living with PCC. Based on patient feedback, we included optional chapters in the manual to reflect their insights and preferences. To improve accessibility, we ensured that the intervention could be delivered online or face‐to‐face, according to patient preference. We also ensured that session durations were optimised to promote meaningful engagement while avoiding fatigue, the most reported symptom in PCC. The complex psycho‐physiological experience of PCC, where physical and psychological elements are inextricably linked, demands an intervention that goes beyond treating symptoms in isolation, towards one that recognises the profound influence each has on one another. Balance‐ACT integrates both physical and psychological strategies to promote both symptom management and improved QoL. We included a comprehensive toolkit to meet these diverse needs, supplemented by flexible scheduling to accommodate patient circumstances. We aimed to foster an inclusive and supportive environment for patients. Balance‐ACT is distinct in its theory‐driven, codeveloped approach, blending ACT with principles of physiological regulation. Our approach attempts to integrate physiological and psychological explanations for individuals with PCC. Balance‐ACT is designed to facilitate psychological flexibility and engagement in meaningful activities but also integrates strategies to enhance physiological functioning. In doing so, Balance‐ACT moves beyond symptom management offering a holistic, therapy‐based model thereby differentiating it from existing approaches. Through the incorporation of PPI, Balance‐ACT caters to the specific needs and lived experiences of individuals with PCC, assuring relevance in real‐world clinical settings.

### 4.1 Strengths and Limitations

A key strength of the development of the intervention was the involvement of people with lived experience, whose ongoing feedback throughout the process was instrumental in continuously refining and tailoring the intervention, ensuring it is well‐suited to the specific needs of the patient population.

The present work presents several limitations. First, a convenience sample was used for the qualitative interviews, which primarily consisted of White British Women in the United Kingdom. Consequently, there are limits in generalising beyond White British patients. Those who took part in the interviews were only presented a preliminary outline of the intervention (as it had not been developed at that stage). This may have influenced their feedback. However, to ensure that the intervention was grounded in the needs of the patients, we received ongoing feedback from several PPI members of our management group. Only a proportion of those interviewed formed the PPI group. Although it included a gender mix, detailed socio‐demographic and clinical information that could have influenced participants' feedback was not collected. The Balance‐ACT model is guided by research in long‐term health conditions but is entirely theoretical at this stage and has not yet been tested in the context of a randomised controlled trial. The comparison of therapists' scores before and after the training was not adequately powered to identify meaningful differences.

### 4.2 Implications for Practice

The findings highlight the need for tailored interventions that address both the physical and psychological challenges associated with PCC. The theoretically coherent and flexible approach developed in this study has the potential to improve patient outcomes and support recovery, but its efficacy must be evaluated before making any specific recommendations for delivery within the NHS. The complex and variable nature of PCC necessitates adaptable interventions and the development of extensive, detailed materials to include all potentially relevant components. We are currently evaluating this approach in a randomised controlled trial.

## 5 Conclusion

Using a theory‐driven approach, we have developed a novel intervention that is acceptable to patients and HCPs. Whilst PCC is complex, and some concepts of Balance‐ACT may be challenging to follow, we have created materials that are clear and actionable, including mindfulness recordings and an animation. These were iteratively developed and refined based on feedback from stakeholders and PPI representatives. PPI members were integral to the development process, offering detailed feedback which helped ensure the intervention was grounded in the needs of the patients. Future evaluation on the intervention will be key to evaluating its efficacy, laying the groundwork for further refinement and evaluation.

## Author Contributions

**Lily Felton:** writing – review and editing, writing – original draft. **Michail Kalfas:** writing – review and editing, writing – original draft. **Debbie Brewin:** conceptualization, supervision, project administration, visualization, writing – review and editing. **Carole Beckwith:** visualization, conceptualization. **Tasbiha Khan:** formal analysis. **Caroline Jolley:** writing – review and editing, conceptualization, visualization, project administration. **Nicholas Hart:** writing – review and editing. **Emma L. Duncan:** writing – review and editing, visualization, conceptualization. **Timothy Nicholson:** writing – review and editing. **Oliver Witard:** writing – review and editing. **Julie Moore:** conceptualization. **Alan Metcalfe:** conceptualization. **Gerrard Rafferty:** conceptualization. **Trudie Chalder:** conceptualization, resources, supervision, project administration, visualization, writing – review and editing, funding acquisition, investigation.

## Disclosure

The author's views are their own and do not necessarily align with those of Guy's and St Thomas' Charity.

## Conflicts of Interest

T.C. is part‐funded by the National Institute for Health Research (NIHR) Biomedical Research Centre at a UK NHS Foundation Trust. Has authored several self‐help books on chronic fatigue; received ad hoc payments for workshops on long‐term conditions and Long COVID; served on the Expert Advisory Panel for COVID‐19 Rapid Guidelines; received travel expenses and accommodation for attending conferences, and is in receipt of additional COVID‐related grants from UKRI. E.L.D. has received funding for research into Long COVID from the Chronic Disease Research Foundation, specifically for the genetics of PCS/ongoing symptomatic COVID‐19. Has also received funding from the BMA Foundation and Guy's and St Thomas' Charity for Long COVID‐related research. C.J. has received funding from the BMA Foundation for research into Long COVID. The other authors declare no conflicts of interest.

## Supporting information

Supporting Information.

## Data Availability

The authors have nothing to report.

## Endnotes

1 Cytokines are small proteins which act as chemical messengers to communicate between cells and regulate inflammation and the immune response [6].

2 Defusion is a technique used to help individuals detach from unhelpful thoughts by viewing them as temporary mental events rather than absolute truths.
