# Supplementary material for: Codeveloping a Novel Intervention for People With Post‐COVID Condition: The Balance‐ACT Study
Source: Health Expect. 2025 Jun 8;28(3):e70320. doi: 10.1111/hex.70320 (PMC12146418; doi:10.1111/hex.70320)
Supplement: Supplementary file 1 — Supporting Information. [file HEX-28-e70320-s001.docx]

**Appendix**

**Table A1.** *Interview guide*

**Feedback on the study intervention (Balance-ACT)**

We have sent you an information sheet with details about the intervention.

1. How acceptable does Balance-ACT seem to you?
2. Do you think it might help people with Long COVID?
3. Does the intervention make sense to you?
4. Do you think Balance-ACT is a safe intervention?
5. How confident are you that people with Long Covid could do things according to the ACT model?
6. What might be the challenges for people using Balance-ACT?
7. What are your thoughts on the session content? What topics might be useful for people with long COVID?
8. How can Balance-ACT be improved and adapted to the needs of people with Long COVID?
9. What could be done to make the study procedures more acceptable to people with Long COVID?

**Table A2.** *Outline of therapists training programme*

| Background to the Balance-ACT Clinical Trial  Definition and predictors of long COVID  Physiological processes  Psychological processes  Balance-ACT model  Maintenance processes and targets for therapy  Introduction to participant handbook  Case example and application of the model |
| --- |

**Table A3.1.** *Demographic characteristics of the patient sample*

| **Participant Number** | **Sex** | **Age** | **Ethnicity** |
| --- | --- | --- | --- |
| P0101 | Female | 50 | White British |
| P0102 | Female | 33 | White British |
| P0103 | Female | 35 | White British |
| P0104 | Female | 55 | White British |
| P0105 | Female | 38 | White Other |
| P0106 | Female | 51 | Black British |
| P0107 | Female | 61 | White European |
| P0108 | Female | 49 | White British |
| P0109 | Female | 30 | White British |
| P0110 | Female | 49 | Other |
| P0111 | Female | 47 | White British |
| P0112 | Male | 59 | White British |
| P0113 | Female | 75 | White British |
| P0114 | Female | 38 | White British |
| P0115 | Male | 78 | White British |
| P0116 | Male | 40 | White British |
| P0117 | Male | 62 | White British |
| P0118 | Male | 54 | White British |
| P0119 | Male | 46 | White British |

**Table A3.2.** *Demographic characteristics of the HCP sample*

| **Participant Number** | **Sex** | **Age** | **Ethnicity** |
| --- | --- | --- | --- |
| P0201 | Female | 29 | White British |
| P0202 | Male | 39 | White British |
| P0203 | Male | 33 | Black African |
| P0204 | Male | 38 | White British |
| P0205 | Male | 40 | White Other |
| P0206 | Male | 43 | Mixed |
| P0207 | Female | 28 | White Other |
| P0208 | Female | 26 | White Other |
| P0209 | Female | 56 | White British |
| P0210 | Female | 40 | Asian British |
| P0211 | Female | 47 | Asian British |
| P0212 | Female | 46 | White Other |

**Table A3.3*.*** *Demographic characteristics of the PPI sample*

| **Participant Number** | **Sex** | **Age** | **Ethnicity** |
| --- | --- | --- | --- |
| PPI1 | Female | 50 | White British |
| PPI2 | Female | 33 | White British |
| PPI3 | Female | 49 | White British |
| PPI4 | Female | 51 | Black British |
| PPI5 | Male | 54 | White British |
| PPI6 | Male | 46 | White British |
| PPI7 | Female | 30 | White British |

**Table A4.** *Patient and HCP interviews: themes and subthemes*

| **Theme** | **Subthemes** | **Quotes** |
| --- | --- | --- |
| **Acceptability** | Potential Benefits/Usefulness | “*I think it would be very useful”* (P0109)  *“I feel like doing something like that would make a huge difference.”* (P0102)  *“finding an intervention which is holistic and it's going to look at the whole person is going to be very useful I think, or potential to be very useful, especially if it's, you know, it's a sort of intervention which hopefully, whatever the underlying issues are should cover, whether it's psychological and physical.”* (P0204)  *“The content I looked at it was it was very good in terms of the different elements of it, in terms of the values of aspect. It was easy to digest, so I thought that was really good” (P02010)*  *“The first time I heard uh the full form of ACT acceptance commitment therapy and I was very much in love with the word acceptance because this is the problem. Acceptance is the problem in life in general.”* (P02011)  *“you know I think it sounds a really, really good opportunity you know to work this way … so acceptance is looking at values, looking at balances I think will be absolutely fantastic.”* (P0209)  *"quite a motivated bunch of individuals", "quite sort of type A personality individuals" (P0202)* |
|  | Perceived Safety | *“A safe intervention? Yeah I think so” (P0109)*  *“there shouldn't be any further scope of distress apart from their symptoms and feeling frustrated and not recovering.”* (P02011) |
|  | Intervention Makes Sense | *“yeah, I guess it makes perfect sense to me” (P0102)*  *“I think showing them that it's there is some evidence based behind it that it really can help”(P0204)*  *“if you were kind of explaining it in a sense of the systems that are involved, it's not like a one system of the just focusing on the physiological. It's about understanding the clockwork of it all in terms of the body. And if they are understood of that the kind of the biology, the, the psychology of it all the like the bio psych model that we use, then they're going to be on board with it.”* (P02010)  *“I think it's [i.e., the intervention] self-paced by patients, led by patient goals and wants and needs.” (P0205)*  *“I'm just really interested in this homeostasis point that you just raised basically, because that's kind of what I've been saying without having that term” (P01016)* |
| **Flexibility of Intervention Delivery** | Accessibility | *“it improves equity of access to interventions. And so that's why it would be nice to be able to measure the primary outcome virtually or remotely and also deliver the intervention remotely as well. So that's really important, I think it sounds good.”* (P0205)  *“There might be the technology side of things, if you're doing things remotely can be a problem. So you're not necessarily shouldn't be a problem with your younger population who used to this sort of thing, but um, you know, people who aren't less used to it, you know”* (P0204)  *“Yeah, I think, um, I think that in person is better because I think that's part of the therapy is getting people out and to show up.”* (P01010) |
| **Barriers and Suggestions for Implementing ACT Intervention** | Heterogeneity/ Catering to Participants Different Needs | *“I suppose the challenge is, but it's always the case, I guess with any health thing, is that our all of our experiences of long COVID are very different.” (P0101)*  *“And it's gonna be different for everybody, isn't it? To some people, it's gonna be more physical. Some people might have some sort of physical impairment from it. A lot of people won't have any objective physical thing you're going to find, but you are going to find a, you know, large psychological components. So um. And differentiating them is very difficult.” (P0204)*  *“the kind of presentation is so broad, you know for some patients they will be very relevant you know. And definitely what I found was that we have some patients who will present with you know, I think you know, different symptoms will be the kind of problem predominant kind of presentation for patients. I think you know quite a lot of what you've covered there is you know can be quite granular, but I think that can be very useful for some patients and totally irrelevant for others. It's quite tricky because, you know, this group is so heterogeneous”* (P0203) |
|  | The Importance of Acknowledging the Reality of the Symptoms | *“I think when you're having very real physical symptoms, when somebody tells you to go to therapy, you think why? how is that going to help my physical symptoms?” (P0102)*  *“I think it's more a case of a challenge depending on the individual of how medically focused they are. You know there could be, there'll be some that will be more medically focused and there'll be some that will be open minded to psychological intervention and would want to engage in relation to that.”* (P02010)  *“Some of the patients, what you find with what you'll find with some is some may not like this because they don't want to be labelled with a psychological problem. Many of them are desperately hunting to prove that they've got some physical problem.” (P02002)*  *“I'm imagine some patients might respond negatively to the conception of having a psychological focus,.” (P02003)*  *“Give them that time to speak and to, you know, as you said, go through the kind of your implementing mindfulness.” (P0208)*  *I'm a massive advocate for therapy, so I was very easily convinced. But when I was ill first time around with their me, I was very resistant. Extremely resistant actually, because I just thought, well, “that's rubbish, that's not going to help me at all.” So I think, that could be, that could be a challenge. (P0102)* |
|  | Acceptance | *“it's very difficult to accept yourself.” (P01014)*  *“I guess it's first acceptance that fatigue is there is probably one of the biggest barriers that we find in that. They weren't like it before, but I am now.”* (P0201)  *“especially specialists would come forth as being experts and they just had, they were completely dismissive of what I went through” (P0105)*  *“The first time I heard uh the full form of ACT acceptance commitment therapy and I was very much in love with the word acceptance because this is the problem. Acceptance is the problem in life in general.” (P02011)* |
|  | Creating Engaging Sessions | *“yes, absolutely. I think, I think that's essential, because I think when you're diagnosed you can kind of, you can kind of get a bit worried about doing any exercise because you think it's going to make you too tired.” (P0102)*  *“I guess like the things that I have wanted is yeah, the core thing about activity versus rest and the sort of daily energy management. Yeah sleep. But then I guess I would want it to cover whatever you feel like you have, there is evidence and expertise to be helpful. Like I don’t really need someone to give me a diet if you don’t know if it’s gonna work.” (P0103)*  *“I think what would help enormously would be to have nutrition advice” “So I think it would have to be a specialised nutrition kind of approach that actually has people with expertise on long COVID, rather than generic nutrition and diet advice because I don't think people have enough….” (P0105)*  *“I think nutrition and sleep hygiene are really important as well.” (P0109)*  *“And they may be maximum for 45 minutes to an hour.” (P0101)*  *“It makes sense in the sense that, um, 60 minutes as opposed to the regular IAPT 30 minutes, 60 minutes makes sense, that’s very good….. And I think twelve makes more realistic sense” (P0106)*  *“I think relaxation is a really good part of it” (P01017).*  *“I guess 10 sessions or so is realistic” (P0107)* |

**Table A5:** *Scoping review: interventions and approaches for PCC*

| **Author** | **Condition** | **Sample Size** | **Study Design** | **PCC-related Symptoms** | **Intervention/**  **Comparison** | **Outcome** |
| --- | --- | --- | --- | --- | --- | --- |
| **PCC Studies** | | | | | | |
| Duñabeitia et al. (2022) | PCC | 73 (after removing incomplete cases) | Feasibility Pilot Study | Cognitive dysfunction | Personalised computerized cognitive training (CCT) intervention vs no comparison group | Self-administered CCT, using gamified cognitive tasks, could effectively improve cognitive dysfunction in individuals with PCC. |
| Kuut et al. (2023) | PCC | 114 | Randomised controlled trial | Fatigue | CBT vs care as usual (CAU) | CBT was effective in reducing fatigue, with positive effect sustained at 6-month follow-up. |
| Hausswirth et al. (2023) | PCC | 34 | Randomised controlled trial | Cognitive dysfunction, Fatigue, Sleep disturbances, Pain | Neuro-Meditation Program vs no intervention vs healthy controls | Neuro-meditation reduces cognitive impairment & improves physical/mental fatigue, muscle/joint pain, sleep, depression/ anxiety. |
| Xia et al. (2022) | PCC | 233 | Systematic review | Fatigue, Exercise intolerance | Pulmonary rehabilitation vs control (no care or education) | Patients undergoing pulmonary rehabilitation showed a reduction in fatigue and increased exercise capacity. |
| Jimeno-Almazán et al. (2021) | PCC | Not specified | Narrative review | Exercise intolerance | Exercise-based treatments vs other treatment options | Exercise interventions significantly improved physical function in PPC (including aerobic capacity, muscle strength, and endurance) as well as enhanced QoL. |
| Satana et al. (2023) | PCC | 70 | Randomised controlled trial | Cognitive dysfunction, Fatigue | Active High Definition- Transcranial direct current stimulation (HD-tDCS) or a sham HD-tDCS | Significant reduction in both cognitive and psychological fatigue for treatment group that was greater than the sham group. |
| Elhamrawy et al. (2023) | PCC | 54 | Randomised controlled trial | Fatigue | Tai Chi group (TC), Aerobic training group (AT), or control group | The AT group showed the biggest reduction in fatigue scores. |
| **Studies on Related Conditions** | | | | | | |
| Tan et al. (2019) | Lung Diseases | 63 | Randomised controlled trial | Breathlessness | Standard care plus a 20-minute mindful breathing session or standard care alone. | Found mindful breathing session is effective in reducing dyspnoea. |
| Mankus et al. (2013) | Generalised Anxiety | 67 | Observational research study | Anxiety, Autonomic dysfunction | Observed existing levels of mindfulness, GA symptoms, and HRV among participants. | For individuals with high generalized anxiety, mindfulness may enhance parasympathetic influences on the heart rate. |
| Rusch et al. (2019) | Clinically significant sleep disturbance | 1654 | Systematic review | Sleep disturbances | Structured mindfulness meditation vs active controls | Mindfulness improves sleep quality in sleep disturbed populations |
| Malouff et al. (2008) | Chronic fatigue syndrome (CFS) | 1371 | Meta-Analysis | Fatigue | Either (a) a treatment with a cognitive and/or behavioral emphasis or (b) a control condition. | Psychological interventions such as CBT could improve fatigue. |
| Williams et al. (2020) | Chronic obstructive pulmonary disease (COPD) | 3,215 | Rapid Review | Fatigue, Breathlessness, Exercise intolerance, Anxiety, Depression | High-intensity CBT vs enhanced usual care/usual care | Psychological interventions such as CBT could improve breathlessness, exercise capacity, QoL, anxiety & depression. |
| Kirk & Axelsen (2020) | Healthy population | 99 | Randomised controlled trial | Autonomic dysfunction | Brief 10-day online-based mindfulness intervention vs an active-control group (music listening) and a non-intervention control group | Mindfulness improves HRV in adults |

**Table A6.** *Summary of Balance-ACT intervention patient-facing content*

| **Participant Handbook Chapters** | **Key Messages/Content** | **Session Objectives** |
| --- | --- | --- |
| Chapter 1: Understanding my Body | Homeostasis (the body’s balancing system) is vital to immune system recovery and well-being.  The Autonomic Nervous System, Heart Rate Variability, Circadian Rhythms are key physiological components and the interplay between these systems affects homeostasis. | - Assessment and engagement - Check understanding of the pre-reading materials and information in the Participant Handbook that they have read so far. - Provide a rationale for the focus of the intervention and introduce the programme - Encourage personalization of materials and recording/notes in the Participant Handbook - Encourage use of diaries - Introduce Mindfulness and complete an exercise in session: Noticing, naming describing |
| Chapter 2: About Me | Ongoing COVID recovery impacts people in many different ways.  Learning skills to improve sleep, breathing patters, activity and exercise, relaxation, healthy nutrition, regulating emotions, increasing social support and reducing stress assists with homeostatic regulation and increases well-being and quality of life. Your diaries show what is happening at your starting point.  ACT helps to:  Focus on the present and what can be changed  Recognise when you are caught up with things you cannot change  Step back from distressing symptoms and take action in the direction of your values.  **Mindfulness skills practice:**  NOTICE  Present moment awareness using multiple senses  Noticing, naming and describing experiences. | - Further assessment and engagement - Evaluate material from monitoring diaries - Check understanding of pre-session reading and exercise (if completed). If not, complete exercise from handbook in session or discuss/cover in some way |
| Chapter 3: What is Important | Exploring values and needs helps clarify what gives life meaning and purpose and what is most important.  Taking action steps in the direction of your values improves the quality of your life.  **Skills practice:**  Recognising when you are acting in line with values and responding appropriately to needs. Noticing when you are distant from values and the effect of this. | - Establish deeper understanding of values and needs - Establish longer-term goals and life areas to focus on - Identify small action steps/goals - Personalise programme and materials to the individual - Encourage mindfulness practice: Noticing, naming, describing and present moment focus - Encourage continued use of diaries/monitoring |
| Chapter 4: Understanding Body Reactions: Stress & Emotions | Information about physiological arousal, the effect on the body and symptoms, the emotion systems and how they affect well-being and the immune system.  Stress triggers, ‘stress signature’ and helpful coping strategies.  Threat/Drive/Soothe and how to balance the emotion systems.  **Skills practice/exercises:**  Taking practical steps to reduce stress where possible by doing things which reduce stress  Mindfulness EMBRACE your experience skills  Stepping back and making room for uncomfortable experiences and symptoms. Broadening focus, attention and perspective.  Increase behaviours which balance the emotion systems.  How to be self-compassionate | - Information on the physiology and psychology of the stress response - Techniques for reducing baseline stress - Understanding of the link between emotional and physical/physiological symptoms - Understanding of the emotion systems and how to balance these by changing behaviour - Encourage use of mindfulness skills and introducing acceptance of discomfort and symptoms (embrace- make room) - Encourage self-care/compassion and self-efficacy |
| Chapter 5: Doing What Matters | Identifying what you are already doing that helps keep you in balance and what you could do more of.  Linking this with your values and needs. Setting values-based goals.  **Skills practice/exercises:**  Evaluate activities in a typical day: Nourishing, Depleting, Important  Identifying activities and actions that bring values to life  Expanding repertoire of helpful activities  Mindfulness Practice: Taking Helpful Actions | - Moving from awareness to taking action - Identifying which activities are nourishing/energising; important; soothing or activating - Increasing activity and expanding the repertoire of activities according to needs and values - Identifying values-based goals linked to life areas |
| Chapter 6:  Choosing Wise Actions | Identifying unhelpful thoughts and thinking patterns and how these lead to unhelpful actions and feelings  Identifying thinking traps and unhelpful reactions  wise mind and balanced reactions  **Skills practice/Exercises:**  Tools to help ‘Unhook’ from thoughts and feelings  The Choice Point: BOLD steps and actions that take you towards values | - Identifying patterns of thoughts and feelings which maintain distress and symptom focus and keep the patient ‘stuck’ - Defusion techniques to ‘unhook’ from difficult symptoms, thoughts and feelings - Use of the ‘Choice Point’ to create a pause, make wise choices towards values-based goals |
| Chapter 7: Healthy Life Habits & Balanced Routines | Identifying healthy habits that support homeostasis and balance  The importance of circadian rhythms and heart rate variability  How to create balanced routines through pacing spacing and prioritising  Reducing extreme behaviours  **Skills practice/exercises:**  Checking in with body clock habits and heart rate variability  Time management  Tips for creating habits | - Establishing healthy habits and focussing on homeostasis - Encourage good habits with sleep, nutrition, energy, physical activity - Establish balance in terms of routine, activity and healthy habits - Scheduling - Addressing boom and bust behaviours - Addressing barriers to maintaining change |
| Chapter 8:  Keep Doing What Works | Summary of sessions  Review of progress towards goals  What do I need to know more about or re-visit?  Maintaining progress | - Review of all skills learned in Part One - Opportunity to revise sessions or sections and introduce more from part two and deal with setbacks - Review progress - Encourage self-efficacy and relapse management - Validate practice and hand-over responsibility for maintaining change |
| Balance-ACT Plan | Work with the participant to complete this so that there is a record of what works for them  Plan for relapses |  |
| **Part Two** |  |  |
| Handout A Balanced Breathing | Information, tips on good breathing  • How to manage symptoms  • Relevant techniques and how good breathing links to Balance and homeostasis |  |
| Handout B: Sleep, Rest & Relaxation | • The importance of sleep. Information, tips and strategies for how to manage sleep problems.  • Relaxation techniques and ways to soothe and involve the parasympathetic nervous system. How this links with Balance and homeostasis. |  |
| Handout C: Increasing Physical Activity and Exercise | Information, tips on how to stabilise activity to establish a balanced routine of physical activity, gradually increasing exercise capacity |  |
| Handout D: Nutrition & Energy | Eating for energy and health. Outline of current research evidence Information on the Eatwell Guide and how to improve your healthy eating habits. |  |
| Handout E: Balancing Stress & Emotions | More ways to reduce stress and balance the emotion systems and let go of or defuse from unhelpful thinking |  |
| Handout F: Information for Partners, Relatives and Friends | Information on long COVID and how best to support a person who is recovering and get help |  |
| Seeking Help and Useful Resources | What to do in an Emergency/Adverse Events  Exploring graded return to work. Information and resources for seeking employment advice and support  References and publications  Useful websites and organisations | Encourage participant to explore and  seek appropriate help  Signpost to relevant websites if  necessary |

| Overview of the Balance-ACT Trial  Understanding long COVID  Introduction to Balance-ACT  Balance-ACT targets and mechanisms for change  Phases of the intervention  Therapeutic techniques in Balance-ACT  Key materials |
| --- |

**Table A7.** *Content of therapist manual*

**Table A8**. *Balance-ACT handbook and manual amendments.*

Changes made to the ACT+ intervention and associated materials as part of the iterative development process include:

| ***Feedback from PPI, TMG and REC*** | ***Implications for the development of the intervention materials and training*** | ***Amendments to Participant Handbook*** | ***Amendments to Therapist Manual/Training/Supervision*** |
| --- | --- | --- | --- |
| Handbook was interesting, relevant and helpful. However, it was long and at times challenging to read.  (PPI, 22 MAY 23) | The manuals need to be shorter and easier to read | The participant handbook was shortened and the font and line spacing was increased. Some information was simplified slightly, broken down into shorter chapters and colour was included. | The therapist manual was shortened and organised to complement the Participant handbook. |
| People did not like the term “Ongoing COVID recovery”, because they felt it diminished the validity of the condition. They are fine with using the terms “Ongoing” and “Recovery” in the handbook, but not as the label of the condition  (PPI, 22 MAY 23) | It is important to use a term for long COVID that is acceptable to patients | The term “ongoing COVID recovery” was removed | The term “ongoing COVID recovery” was removed |
| Inclusion of emotional aspects of dealing with the condition and the personalisation of the approach was commended but needs careful handling in terms of interpersonal skills  (PPI, 22 MAY 23) | Recruitment selection included requirement for strong interpersonal skills | Amendments to some exercises to ensure they are introduced by Therapists rather than self-guided. | - Interpersonal skills focussed on during training and supervision - Trainer/Supervisor ensures therapists select information, techniques and exercises appropriate to the individual |
| Fatigue and cognitive challenges such as memory loss, concentration difficulties may interfere with ability to process information. Multi-media presentations may be helpful or an audio version of the Participant Handbook.  (PPI, 22 MAY 23) REC Dec 23 | As far as possible there should be fully accessible and multimedia options for dissemination of information | - Read Aloud function available for digital copies of the handbook and available if necessary - Illustrations added to participant handbook - Animation developed to explain the physiological/psychological aspects (homeostasis) - Mindfulness exercises recorded by a PPI member | Animations and Mindfulness Exercises made available for Therapists to use prior to client engagement and as part of the delivery of the intervention. |
| Simplify the language in the booklet and explain any necessary jargon in lay terms.  REC Dec 23  PPI May 23 | There was slight conflict with the REC requirement to simplify and become jargon-free and the PPI feedback which encouraged and endorsed the inclusion of factual research information and terms. | - Glossary of key terms included with jargon-free explanations of medical, physiological or psychological terms - Re-checked with PPI | - No changes to therapist manual or training. - Supervision guidance encouraged a flexible and personalised approach, providing information in a way which the participant identified with and understood without being overly didactic or patronising |
| In conjunction with your PPI group, consider making the following changes to the booklet:   - Split the booklet into chapters and ensure any tables included are be edited so they are on a single page. - Separate out the exercises from the booklet and issue these in increments to the participants. - Add additional space for participant reflections. - Instead of using the scientific terms for the nervous systems throughout the booklet, consider using lay terms. - Part 2 of the booklet states that these sections will be given out if the therapist deems that it would be useful. Clarify if this will be documented so that a record is kept of what extra sections were given and why. - The information for partners and friends in the booklet cold be separated. Clarify if you will document if this is given out and whether you ask for any feedback regarding how helpful it was for the partner/carer/friend or participant themselves. | Aim was to provide an appealing but comprehensive document in workbook format which would not appear overwhelming | - Some exercises removed from the participant handbook and placed in the therapist manual to be used as required - Participant handbook divided into Part 1 and Part 2 with the ‘Information for Family and Supporters’ in Part 2 as supplementary Information - Both Parts 1 and 2 given to all participants but introduced by the therapist during session 1 face to face and explanation on how to use both parts given - Coloured tabs added to separate chapters and sections - More space added for Participant reflections and to use as a ‘workbook’ with notes - Key terms section included - Animation developed to provide information in a more fun, memorable, multi-media format - Final participant handbook printed May 24 and bound in metal spiral binding with coloured tabs to enable ease of handling without too much bulk and weight | - Additional practice sessions added to the therapist training - More comprehensive Session Tips added - Close supervision and ‘Fidelity Feedback’ tool used after first few sessions delivered - Weekly group supervision to supplement training and support treatment integrity and effective delivery of sessions. |

**Table A9.** *Balance-ACT fidelity measure feedback and scoring sheet*

**HCP: Participant ID:**  **Session No: Length of Session:**

| **Item** | **Score** | **Comment/Evidence (Time on recording)** |
| --- | --- | --- |
| **General clinical/interpersonal skills & ACT consistent therapist stance:** | | |
| **G1. Structure, pacing, timing and organisation** (uses strategies to ensure effective use of time and support session objectives, follows a collaborative agenda) |  |  |
| **G2. Interpersonal style and therapeutic alliance** (supportive encouragement, understanding, warmth, empathy, collaborative style) |  |  |
| **G3. Collaborative, dynamic formulation and therapy plan (**Gathers feedback/relevant information from assessment and ongoing interactions, shared understanding of maintaining factors and targets of the intervention) |  |  |
| **G4. Therapist chooses methods that are sensitive to the situation and context** (i.e. in a flexible and responsive way rather than a 'one size fits all' approach. Interactive information-giving relevant to participant understanding and needs). |  |  |
| **G5. Therapist uses experiential methods/questions**  (i.e. helps the client to notice and use their own experience rather than thoughts about their experience). |  |  |
| **G6. Therapist demonstrates/models willingness & acceptance** (conveys that it is natural to experience difficult thoughts, feelings and symptoms when one is in circumstances such as those experienced by the client and is able to sit with/explore these). |  |  |
| **Open response style** | | |
| **A1. Therapist helps the client to notice thoughts as separate experiences from the events they describe**. |  |  |
| **A2. Therapist gives the client opportunities to notice how they interact with their thoughts, feelings and/or symptoms** (e.g. whether avoidant or open). |  |  |
| **A3. Therapist encourages the client to accept “stay with” or be willing to experience unwanted thoughts, feelings or symptoms in the service of their values** |  |  |
| **Aware response style** |  |  |
| **A4. Therapist uses present moment focus methods to increase awareness of the moment, including thoughts and feelings.** (e.g. mindfulness tasks, tracking, noticing, etc.) |  |  |
| **A5. Therapist helps the client to notice the stimuli that hook them away from the present moment** (thoughts, feelings, situations, symptoms etc) |  |  |
| **A6. Therapist helps the client to experience that they are bigger than and/or separate from their psychological experiences**. |  |  |
| **Active response style** | | |
| **A7. Therapist gives the client opportunities to notice workable and unworkable responses** (e.g. do their actions move them towards or away from values) |  |  |
| **A8. Therapist gives the client opportunities to clarify their own values** (overarching life goals and qualities of action) |  |  |
| **A9. Therapist helps the client to make plans and set goals likely to meet reinforcing consequences**  (shapes action that is consistent with values) |  |  |
| **Balance-ACT specific interventions** | | |
| **B1. Therapist delivered therapy consistent with the Balance-ACT intervention logic model** (addressed regulation of sleep, activity, stress and emotions) |  |  |
| **B2. Therapist supported routines, long-term habit formation and behaviour change in line with Balance-ACT targets** |  |  |
| **B3. The intervention delivered aimed to restore balance physiologically and psychologically** |  |  |

| **General Comments/Feedback** | |
| --- | --- |
|  | |
| **Rater ID:** | **Date:** |

0 = This behaviour never occurred but was relevant or necessary for the session

(NB score N/A 2 if the behaviour did not occur but was not relevant, necessary or appropriate)

1 = Therapist rarely enacts this behaviour

2 = Therapist sometimes enacts this behaviour

3= Therapist consistently enacts this behaviour

4= Therapist enacts this behaviour consistently and demonstrates a high degree of skill

| Item Group | Score | Competency Range |
| --- | --- | --- |
| General Clinical/Interpersonal skills & ACT Stance G1-G6 |  | 12- 18 |
| ACT Specific Competencies A1-A9 |  | 18 – 27 |
| Balance-ACT Specific Competencies B1-B3 |  | 6- 9 |
| **TOTAL Scores** |  | 36 - 54 |

**References**

Duñabeitia, J. A., Mera, F., Baro, Ó., Jadad-Garcia, T., & Jadad, A. R. (2023). Personalized computerized training for cognitive dysfunction after COVID-19: a before-and-after feasibility pilot study. *International Journal of Environmental Research and Public Health*, *20*(4), 3100.

Elhamrawy, M. Y., Mohammad el Sherbini, A., Mokhtar, M. M., Mashaal, A., Elkady, S. M., Esladany, S. M., & Said, M. T. (2023) Effect of Tai Chi versus Aerobic Training on Improving Hand Grip Strength, Fatigue, and Functional Performance in Older Adults Post-COVID-19: a

randomized controlled trial. *Journal of Population Therapeutics & Clinical Pharmacology,* 30(7), 190-198. Doi: 10.47750/jptcp.2023.30.07.024

Hausswirth, C., Schmit, C., Rougier, Y., & Coste, A. (2023). Positive impacts of a four-week neuro-meditation program on cognitive function in post-acute sequelae of COVID-19 patients: a randomized controlled trial. *International journal of environmental research and public health*, *20*(2), 1361.

Jimeno-Almazán, A., Pallarés, J. G., Buendía-Romero, Á., Martínez-Cava, A., Franco-López, F., Sánchez-Alcaraz Martínez, B. J., ... & Courel-Ibáñez, J. (2021). Post-COVID-19 syndrome and the potential benefits of exercise. *International journal of environmental research and public health*, *18*(10), 5329.

Kirk, U., & Axelsen, J. L. (2020). Heart rate variability is enhanced during mindfulness practice: A randomized controlled trial involving a 10-day online-based mindfulness intervention. *PloS one*, *15*(12), e0243488.

Kuut, T. A., Muller, F., Csorba, I., et al. (2023). Efficacy of cognitive-behavioral therapy targeting severe fatigue following coronavirus disease 2019: Results of a randomized controlled trial. Clinical Infectious Diseases, 77(5), 687-695. https://doi.org/10.1093/cid/ciad257

Malouff, J. M., Thorsteinsson, E. B., Rooke, S. E., Bhullar, N., & Schutte, N. S. (2008). Efficacy of cognitive behavioral therapy for chronic fatigue syndrome: a meta-analysis. *Clinical psychology review*, *28*(5), 736-745.

Mankus, A. M., Aldao, A., Kerns, C., Mayville, E. W., & Mennin, D. S. (2013). Mindfulness and heart rate variability in individuals with high and low generalized anxiety symptoms. *Behaviour research and therapy*, *51*(7), 386-391.

Rusch, H. L., Rosario, M., Levison, L. M., Olivera, A., Livingston, W. S., Wu, T., & Gill, J. M. (2019). The effect of mindfulness meditation on sleep quality: a systematic review and meta-analysis of randomized controlled trials. *Annals of the New York Academy of Sciences, 1445*(1), 5–16. https://doi.org/10.1111/nyas.13996

Santana, K., Franca, E., Sato, J., Queiroz, M., Farias, J., Rodrigues, D., Souza, I., Ribeiro, V., Caparelli-Daquer, E., Teixeira, A. L., Charvet, L., Datta, A., Bikson, M., & Andrade, S. (2023). Non-invasive brain stimulation for fatigue in post-acute sequelae of SARS-CoV-2 (PASC). *Brain stimulation,* 16(1), 100-107. Doi: 10.1016/j.brs.2023.01.1672

Tan, S. B., Liam, C. K., Pang, Y. K., Ng, D. L. C., Wong, T. S., Khoo, K. W. S., ... & Chai, C. S. (2019). The effect of 20-minute mindful breathing on the rapid reduction of dyspnea at rest in patients with lung diseases: a randomized controlled trial. *Journal of pain and symptom management*, *57*(4), 802-808.

Williams, M. T., Johnston, K. N., & Paquet, C. (2020). Cognitive behavioral therapy for people with chronic obstructive pulmonary disease: rapid review. *International Journal of Chronic Obstructive Pulmonary Disease*, 903-919.

Xia, R., Zhang, Q., Chen, J., Zhang, Y., Zhang, Z., & Li, H. (2022). The effect of pulmonary rehabilitation on patients with post-COVID-19 syndrome: A systematic review and meta-analysis. Frontiers in Medicine, 9, 837420. https://doi.org/10.3389/fmed.2022.837420
